# Fabrication of Antibacterial and Antioxidant ZnO-Impregnated Amine-Functionalized Chitosan Bio-Nanocomposite Membrane for Advanced Biomedical Applications

**DOI:** 10.3390/molecules28207034

**Published:** 2023-10-11

**Authors:** Ali M. Ali, Abdelrahman M. Hamed, Mahmoud A. Taher, Mohamed H. Abdallah, Mohamed Abdel-Motaleb, Zyta M. Ziora, Ahmed M. Omer

**Affiliations:** 1 Chemistry Department, Faculty of Science, AL-Azhar University, Assiut 71524, Egypt; aly.aly88@yahoo.com (A.M.A.); abdo8522@yahoo.com (A.M.H.); mahmoudtaher@yahoo.com (M.A.T.); mohamed.hassan14@azhar.edu (M.H.A.); tolba1950@hotmail.com (M.A.-M.); 2 Institute for Molecular Bioscience, University of Queensland, St. Lucia, Brisbane, QLD 4072, Australia; 3 Polymer Institute of the Slovak Academy of Sciences, Dúbravská Cesta 9, 845 41 Bratislava, Slovakia; 4 Polymer Materials Research Department, Advanced Technology and New Materials Research Institute (ATNMRI), City of Scientific Research and Technological Applications (SRTA-City), New Borg El-Arab City, Alexandria 21934, Egypt

**Keywords:** aminated chitosan, ZnO nanoparticles, antibacterial activity, antioxidant activity, biodegradable membrane

## Abstract

Developing a variety of safe and effective functioning wound dressings is a never-ending objective. Due to their exceptional antibacterial activity, biocompatibility, biodegradability, and healing-promoting properties, functionalized chitosan nanocomposites have attracted considerable attention in wound dressing applications. Herein, a novel bio-nanocomposite membrane with a variety of bio-characteristics was created through the incorporation of zinc oxide nanoparticles (ZnONPs) into amine-functionalized chitosan membrane (Am-CS). The developed ZnO@Am-CS bio-nanocomposite membrane was characterized by various analysis tools. Compared to pristine Am-CS, the developed ZnO@Am-CS membrane revealed higher water uptake and adequate mechanical properties. Moreover, increasing the ZnONP content from 0.025 to 0.1% had a positive impact on antibacterial activity against Gram-positive and Gram-negative bacteria. A maximum inhibition of 89.4% was recorded against *Escherichia coli*, with a maximum inhibition zone of 38 ± 0.17 mm, and was achieved by the ZnO (0.1%)@Am-CS membrane compared to 72.5% and 28 ± 0.23 mm achieved by the native Am-CS membrane. Furthermore, the bio-nanocomposite membrane demonstrated acceptable antioxidant activity, with a maximum radical scavenging value of 46%. In addition, the bio-nanocomposite membrane showed better biocompatibility and reliable biodegradability, while the cytotoxicity assessment emphasized its safety towards normal cells, with the cell viability reaching 95.7%, suggesting its potential use for advanced wound dressing applications.

## 1. Introduction

Modern wound dressings have recently been used instead of traditional dressings because they provide a moist environment for the wound area and accelerate the relocation of epithelial cells to replace the dead cells and rebuild the damaged tissue [1,2]. The most important advantages of modern wound dressings are their simplicity of application, ease of sterilization, inhibition of bacterial attack, reduction of wound inflammation, biodegradability, and acceptable mechanical properties [3]. Lately, natural biopolymers have been expansively employed in countless biomedical applications owing to their outstanding properties comprising their availability in nature, eco-friendliness, biodegradability, non-toxicity, and beneficial biological characteristics [4,5]. Among them, chitosan (CS) is the deacetylated form of chitin biopolymer, and it comprises randomly positioned glucosamine and *N*-acetyl glucosamine units linked by *β*-(1–4) glycosidic bonds [6,7]. Owing to its intriguing biological properties, CS has been broadly engaged in various medical and pharmaceutical fields, including wound healing, tissue engineering, drug and gene delivery [8,9,10,11]. CS can stimulate the growth of fibroblasts, halt bleeding, and motivate the migration of both mononuclear and poly-morphonuclear cells, which consequently improves re-epithelization and skin tissue regeneration [12]. Also, CS possesses adequate antioxidant, anti-cancer, antifungal, and antimicrobial activities [13]. Although CS showed potential activity against a wide-range of microorganisms, this activity has become repressed due to the constant mutations of microorganisms to repel the action of antibiotics [14]. Thus, plentiful physical and chemical modification techniques have been performed on pristine CS to overcome this limitation, such as crosslinking, Schiff base formation, grafting, quaternization, carboxy methylation, and composite formation [8,15,16,17,18].

ZnONPs are a promising and versatile inorganic material extensively applied in biomedical applications owing to their unique characteristics, such as cost-effectiveness, ease of fabrication, high thermal stability, large surface area, high catalytic activity, and non-toxicity [19,20]. ZnONPs demonstrate auspicious inhibition activity against both Gram-positive and Gram-negative bacteria. However, the mechanism of this activity is still scarcely known. Prior reports proposed that this activity involves its accumulation in the outer membrane of bacterial cells, resulting in the cell membrane breakdown [21]. Additionally, ZnONPs can interact with the bacterial core and enter the cell, offering distinct bactericidal mechanisms [22,23]. In addition, it also establishes respectable antioxidant activity since they can induce extra ROS generation, such as superoxide anions, OH^-^radicals, and H_2_O_2_ production [24].

Several studies have recently investigated the impact of the combination of CS and ZnONPs on their antimicrobial and antifungal activities [25,26]. Likewise, electrospun ZnO-loaded chitosan/PCL bilayer membranes have been designed for accelerated wound healing [27]. However, the combination of amine-functionalized chitosan derivative (Am-CS) and ZnONPs for antibacterial and antioxidant wound dressing applications has not yet been studied. An attempt was made in this investigation to create ZnO@Am-CS bio-nanocomposite membranes with multiple predicted bio-characteristics, allowing their unique properties to be combined. To further enhance the antibacterial and antioxidant properties of the original CS biopolymer, the Am-CS derivative was initially synthesized with extra amine groups in addition to its primary active amine group. It is anticipated that adding ZnONPs to the Am-CS membrane will give bio-nanocomposite membranes improved mechanical and roughness properties in addition to the multiple bio-characteristics. The developed ZnO@Am-CS membranes were thoroughly characterized by their chemical structure, morphology, and elemental composition using various characterization tools. Additionally, the impact of the ZnONP ratio on the antibacterial activity of bio-nanocomposite membranes was studied against both Gram-positive and Gram-negative bacteria, while the antioxidant activity was also examined. Furthermore, their in vitro properties, including their hemocompatibility, biodegradability, and cytotoxicity, were also evaluated.

## 2. Results and Discussion

### 2.1. FT-IR Analysis

**Figure 1:**
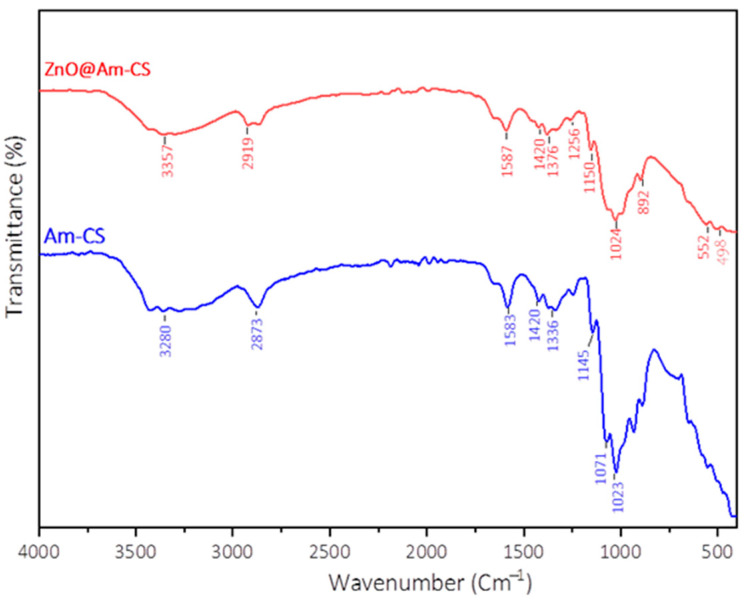
FT-IR spectra of pure Am-CS and ZnO@Am-CS bio-nanocomposite membranes.

Figure 1 displays the IR spectra of pristine Am-CS and ZnO@Am-CS bio-nanocomposite membranes. The observed absorption band at 3280 cm^−1^ in the case of Am-CS signifies the stretching vibration of the -NH_2_ and -OH groups [28,29]. This band was shifted after the formation of the ZnO@Am-CS bio-nanocomposite to a higher wavenumber of 3357 cm^−1^. Also, the stretching vibration bands at 2919 and 2973 cm^−1^ are associated with the C-H aliphatic [30]. Moreover, the absorption bands at 1618 and 1583 cm^−1^ are related to C=O stretching of the secondary amide [8]. The detected bands at 1363 and 1376.14 cm^−1^ correspond to -C=N- stretching and C-H symmetric bending of the CH_2_ group on the polysaccharide skeleton. Further, the skeletal vibrations at 892 and 1023 cm^−1^ correspond to the C-C, C-O, and C-O-C glycosidic bonds [10]. Notably, the observed absorption peaks at approximately 498–600 cm^−1^ in the case of ZnO@Am-CS bio-nanocomposite are assigned to ZnO vibrations [31], which confirm the successful impregnation of ZnONPs in the Am-CS membrane matrix.

### 2.2. Morphological and EDX Analysis

**Figure 2:**
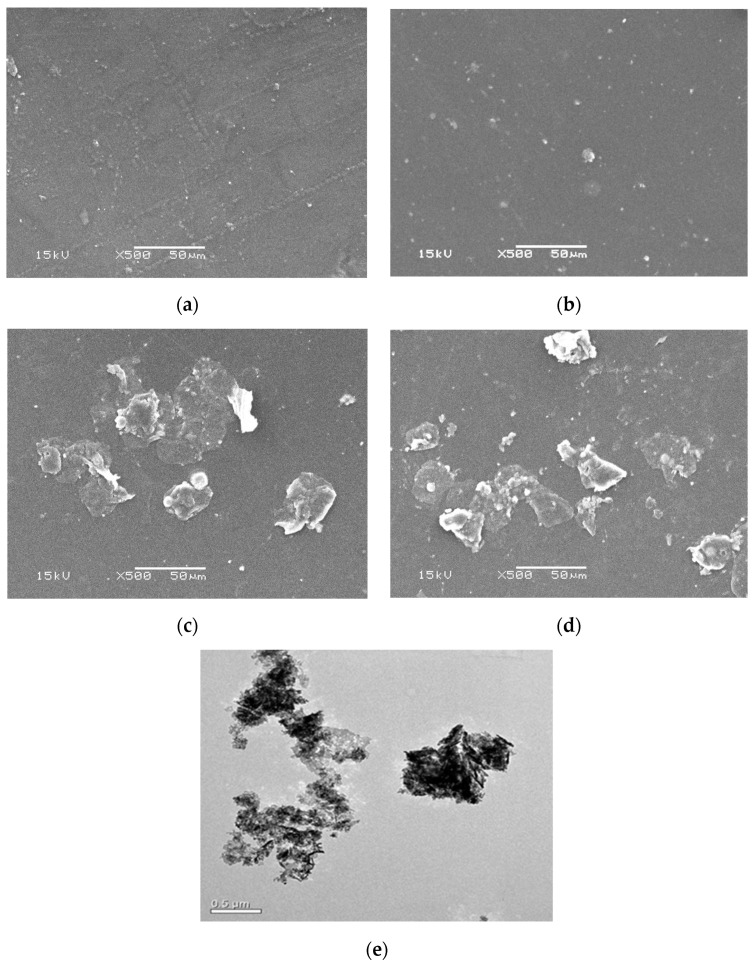
SEM images of (**a**) pure Am-CS membrane, (**b**) ZnO (0.025%) @Am-CS, (**c**) ZnO (0.05%) @Am-CS, and (**d**) ZnO (0.1%) @Am-CS bio-nanocomposite membranes. (**e**) TEM image of ZnONPs at 0.5 µm.

SEM analysis was used to detect the morphological variations induced by the integration of ZnONPs into the membrane matrix of the Am-CS. The SEM image of the native Am-CS membrane (Figure 2a) displayed a relatively coarse and heterogeneous surface [8]. The developed ZnO@Am-CS bio-nanocomposite membranes (Figure 2b–d) showed a rougher surface containing small particles, probably due to the distributed ZnONPs along the Am-CS matrix. In addition, increasing the ZnONP ratio from 0.025% to 0.1% significantly increased the surface roughness, which could be attributed to the polarity difference between them. Likewise, the generated interactions between the Am-CS and the ZnONPs affected the surface morphology of the final bio-nanocomposite membranes. Additionally, TEM analysis (Figure 2e) was performed to gain more details regarding the crystallographic and morphological properties of ZnONPs with high spatial resolution. The ZnONPs displayed a lattice structure with clustered spheroids, which existed in aggregated forms with a slight variation in thickness. This could be ascribed to the high electron density of the ZNPs and the extensive hydrogen bonding between the NPs [32,33,34,35].

**Figure 3:**
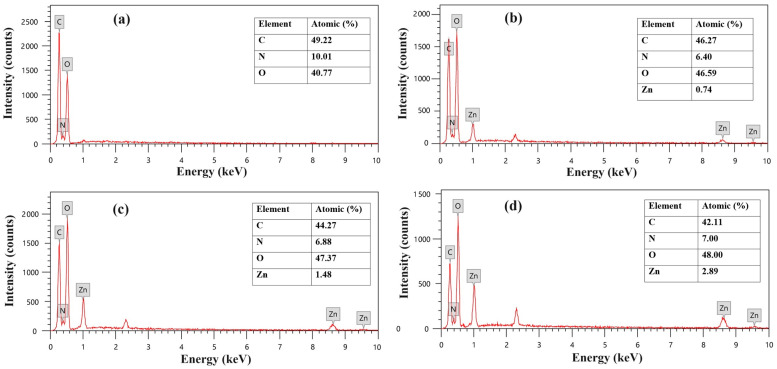
EDX spectra of (**a**) pure Am-CS membrane, (**b**) ZnO (0.025%) @Am-CS, (**c**) ZnO (0.05%) @Am-CS, and (**d**) ZnO (0.1%) @Am-CS bio-nanocomposite membranes.

Additionally, energy dispersive X-ray (EDX) analysis was performed to investigate the elemental composition of the developed membranes, as shown in Figure 3. The results verify that the pure Am-CS membrane (Figure 3a) was composed of C, N, and O with atomic percentages of 49.22, 10.01, and 40.77%, respectively. The incorporation of ZnONPs in the membrane matrix was verified by the existence of a Zn peak in the spectra, while the atomic percentage of Zn was increased from 0.74 to 2.98% with an increasing ZnONP ratio from 0.025 to 0.1% in the composite membrane (Figure 3b–d). Similar observations have been reported by other researchers [36]. These findings infer the successful formation of ZnO@Am-CS bio-nanocomposite membranes.

### 2.3. Mechanical Properties

The mechanical properties of the formulated membranes were also studied, as depicted in Table 1. The results clarify that by increasing the ZnONP content in the ZnO@Am-CS bio-nanocomposite membrane matrix, the mechanical properties were improved. An increase in the tensile strength was perceived with an increasing ZnONP percentage from 0.025% to 0.1%. Therefore, the maximum force of 147 ± 1.2 N, maximum stress of 64.4 ± 1.3 N/m^2^, and maximum strain of 17.5 ± 1.5% were recorded in the ZnO (0.1%) @Am-CS bio-nanocomposite membrane compared to 120.2 ± 1.3 N, 42.1 ± 2.1 N/m^2^, and 6.3 ± 1.1% for the pure Am-CS membrane (Table 1). The enhancement of all mechanical parameters with increasing ZnONPs could be due to their presence between the Am-CS polymer chains since the intermolecular crosslinking effect could be generated [31]. Consequently, the formation of manifold intramolecular and intermolecular hydrogen bonds between the Am-CS functional groups (NH_2_ and OH) and the ZnONPs caused a rise in membrane rigidity.

### 2.4. Surface Roughness

**Figure 4:**
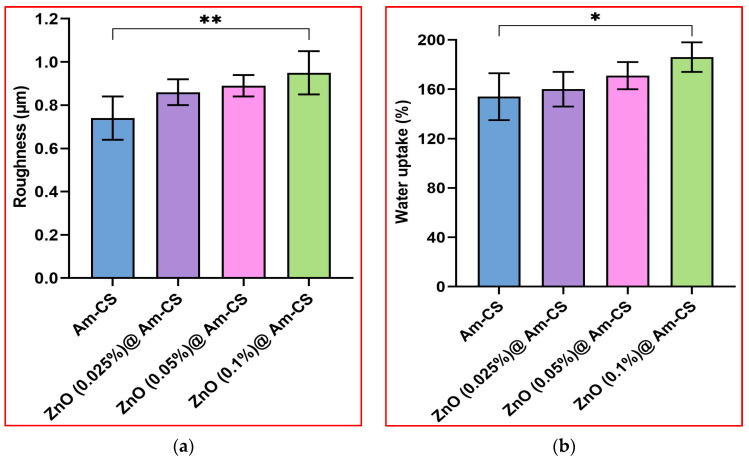
(**a**) Surface roughness and (**b**) water uptake (%) values of pure Am-CS membrane and ZnO@Am-CS bio-nanocomposite membranes. All measurements are presented in five replicates (*n* = 5), and the data are expressed as the mean standard deviation ± SD. (** *p* < 0.01 and * *p* < 0.05).

Figure 4a parades the impact of the ZnONP ratio on the surface roughness of the formulated ZnO@Am-CS bio-nanocomposite membranes. It is clear that the roughness meaningfully increased with an increasing presence of ZnONPs in the membrane matrix. Therefore, the native Am-CS recorded a minimal roughness value of 0.77 ± 0.14 μm, while the surface roughness of bio-nanocomposite membranes recorded maximal values of 0.83 ± 0.13 μm with 0.025% ZnONPs, 0.88 ± 0.16 μm with 0.05% ZnONPs, and 0.95 ± 0.12 μm with 0.1% ZnONPs. These results agree with those obtained by SEM analysis. Increasing the surface roughness improved the adhesion ability of the membrane to the tissue, and as a result, the cell attachment can be enhanced, confirming its use as an efficient antibacterial wound dresser [37].

### 2.5. Water Uptake Profiles

The water uptake property is considered one of the most vital characteristics of wound dressing membranes since it provides a moist environment for the wound area and simplifies the passage of fibroblasts, keratinocytes, and endothelial cells to the injured area. Additionally, this property can help in the absorption of surplus wound exudates, boost the hemostasis property, and hence, accelerate the wound healing process [8,38]. Figure 4b shows the water uptake profiles of pure Am-CS and its nanocomposite. Hydrophilic OH and NH_2_ groups in the membrane matrix facilitate binding with water molecules, resulting in a high-water uptake capacity. Interestingly, the ZnO@Am-CS bio-nanocomposite membranes enhanced water uptake behavior compared to the pure Am-CS. Therefore, increasing the amount of ZnONPs in the membrane matrix from 0.025 to 0.1% significantly increased the water uptake value from 160 ± 0.12 to 184 ± 0.17%, while the Am-CS membrane recorded the lowest value of 154 ± 0.15%. It is well known that ZnO is a hygroscopic material that is morphologically rough, which provides a greater number of adsorption sites for water molecules [39]. It has been reported that the surface of a ZnO single crystal is intrinsically hydrophilic [40], and hence, water molecules may adhere onto the ZnO surface without any chemical reaction, providing more hydrophilicity to the bio-nanocomposite membranes [34,41].

### 2.6. Bio-Evaluation Studies

#### 2.6.1. Evaluation of Antibacterial Activity

**Figure 5:**
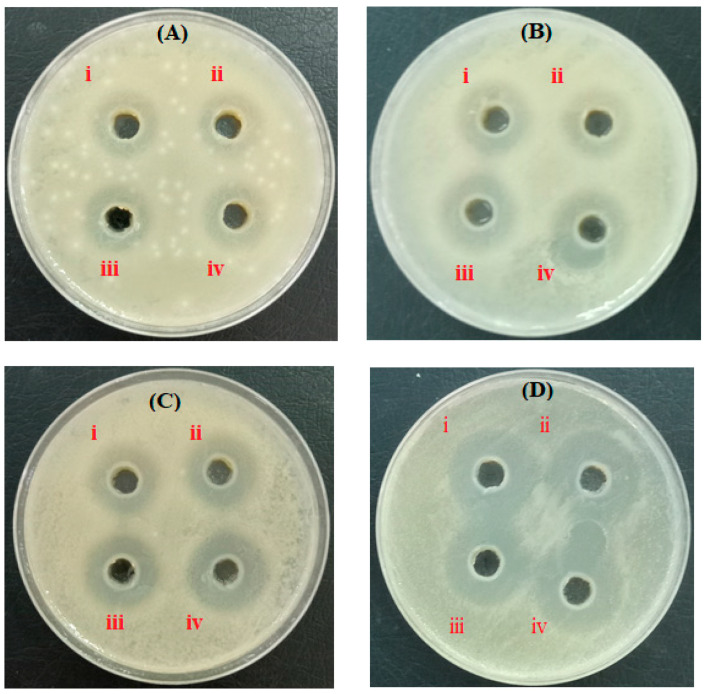
Images of inhibition zones of (**A**) *S. haemolyticus*, (**B**) *K. pneumoniae*, (**C**) *P. aeruginosa*, and (**D**) *E. coli* for (**i**) Am-CS, (**ii**) ZnO (0.025%) @Am-CS, (**iii**) ZnO (0.05%) @Am-CS, and (**iv**) ZnO (0.1%) @Am-CS bio-nanocomposite membranes. All values are presented in five replicates (*n* = 5), and the data are expressed as the mean standard deviation ± SD.

**Figure 6:**
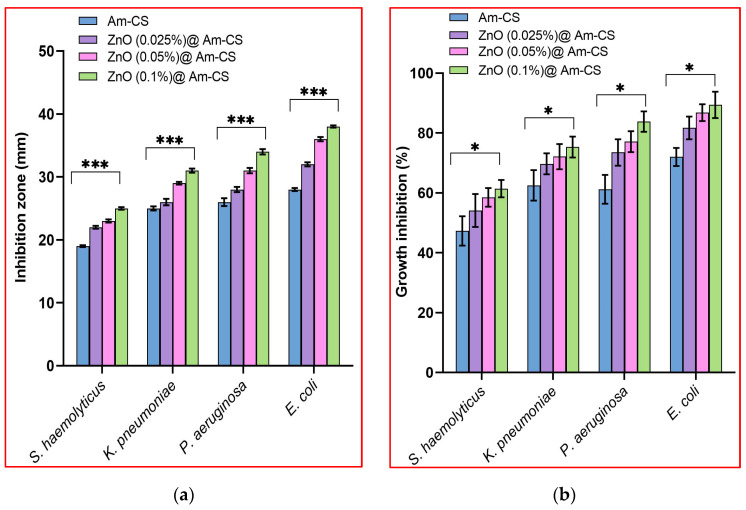
(**a**) Inhibition zones (mm) and (**b**) growth inhibition (%) by Am-CS and its bio-nanocomposite with different ZnO concentrations using the broth dilution method. All values are presented in five replicates (*n* = 5), and the data are expressed as the mean standard deviation ± SD. (*** *p* < 0.001 and * *p* < 0.05).

Bacterial infection delays the process of wound healing, sometimes leading to patient death. Thus, it is indispensable to develop new effective materials for inhibiting the growth of pathogenic bacteria. Following the agar well diffusion method, the antibacterial properties of both pure Am-CS membranes and ZnO@Am-CS bio-nanocomposite membranes were tested. Images of the inhibition zones are depicted in Figure 5. The results imply that all tested membranes showed remarkable antibacterial activity against both types of bacteria. The inhibition zone in the case of Gram-negative bacteria was found to be higher than that of Gram-positive bacteria. It was also observed that increasing the ZnONP percentage in the formulated bio-nanocomposite membranes significantly enhanced the inhibition process. Therefore, the highest inhibition zone diameter of 38 ± 0.17 mm was observed against *E. coli* Gram-negative bacteria and obtained by the ZnO (0.1%) @Am-CS membrane, compared to 28 ± 0.23 mm, which was attained by the native Am-CS membrane (Figure 6a). Similar observations have been reported by other authors, since they concluded that composite chitosan/ZnONPs hydrogel had strong effects on *E. coli* and *S. aureus*, with a higher antimicrobial impact on *E. coli* [35].

The broth dilution assay showed the same trend, since the bio-nanocomposite membranes developed stronger antibacterial potency than Am-CS, as shown in Figure 6b. These results clarify that increasing the ZnONP concentration from 0.025 to 0.1% increased the bacterial inhibition from 54.1 to 61.4% (for *S. haemolyticus*), from 60.7 to 75.3% (for *K. pneumoniae*), from 73.5 to 83.8% (for *P. aeruginosa*), and from 81.7 to 89.4% (for *E. coli*), while the Am-CS recorded inhibitions of 52.3, 66.5, 65.2, and 72%, respectively. The higher antibacterial activity of the formulated bio-nanocomposite membranes could be attributed to both the ZnONPs and Am-CS. It has been reported that ZnO unveils substantial antibacterial activities in the nanostructure, owing to its high specific surface area. ZnONPs can contact cell walls directly and destroy bacterial cell integrity [42,43]. By other means, it can adhere to the surface of bacteria and/or the bacterial core, enter the cell, and exhibit distinct bactericidal mechanisms [21]. Further mechanisms could occur via the creation and gathering of reactive oxygen species (ROS) with oxidative potential, by triggering the destruction of the DNA and bacterial proteins. Additionally, the antibacterial activity may involve the accumulation of ZnONPs in the bacterial cells’ superficial cell membrane or cytoplasm and trigger the release of Zn^2+^ (which acts as antibacterial ions). The discharged Zn ions can electrostatically interact with the bacterial cell membrane. Consequently, this can cause bacterial cell membrane breakdown, membrane protein mutilation, and genomic instability, resulting in the death of bacterial cells. All these mechanisms could cumulatively contribute to the bacterial cell death phenomenon [44].

The antibacterial potency of chitosan derivative (i.e., Am-CS) can also be explained by various mechanisms. The most appropriate one for elucidating this activity involves the electrostatic interactions between the positively charged amino groups of Am-CS and the negative charges on the surface of the bacterial cell wall of Gram-negative bacteria [45,46]. This consequently provokes the escape of intracellular ingredients, such as amino acids, proteins, and glucose, due to disrupting the bacterial cell membrane. Moreover, these interactions could potentially block the feeding channels responsible for exchanging electrolytes and nutrients, where the inhibition process occurs, followed by the death of the bacterial cells accordingly [8,47]. This mechanism explains the effectiveness and the more pronounced antimicrobial potential of ZnO@Am-CS bio-nanocomposite membranes against Gram-negative bacteria in specific *E. coli* compared to Gram-positive bacteria, as the latter possess a membrane with denser layers of peptidoglycan [48] with crosslinked chains of peptidoglycan, creating stiff cell walls by the action of the bacterial enzyme DD-transpeptidase. Thus, the inhibition process of Gram-positive bacteria could be hindered [49].

#### 2.6.2. Evaluation of Antioxidant Activity

**Figure 7:**
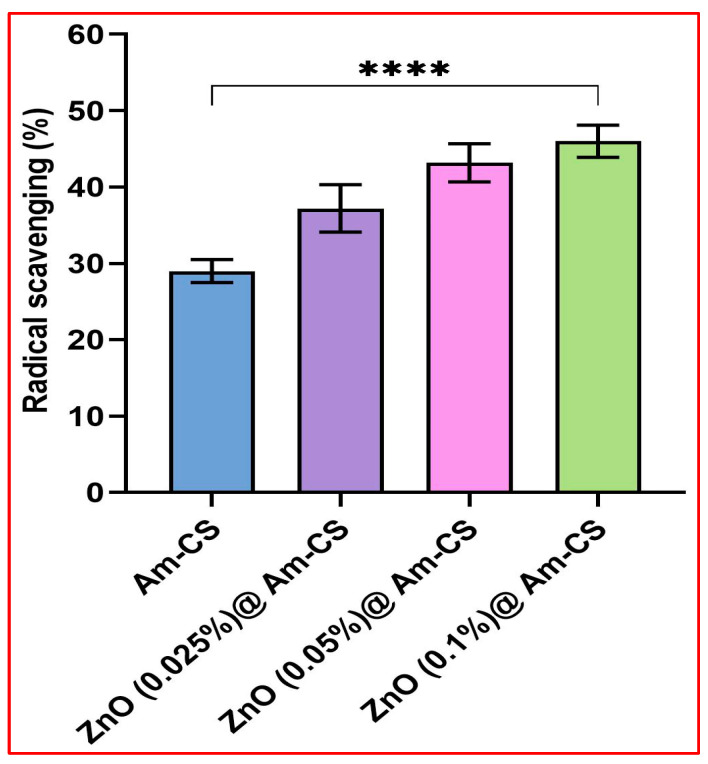
Antioxidant activities of Am-CS and its bio-nanocomposite with different ZnO concentrations using the ABTS assay. All measurements are presented in five replicates (*n* = 5), and the data are expressed as the mean standard deviation ± SD. (**** *p* < 0.0001).

The extreme formation of reactive oxygen species (ROS) in the human body through metabolic processes causes cellular damage to some cells [50]. Therefore, antioxidant materials act as free radical scavengers, which can decrease the probability of chronic disease progression if the dosage is properly designed. This assay is based on an indicator of the capability of antioxidants to scavenge ABTS and produce ABTS^•+^, which acts as an agent for hydrogen donation [51]. ABTS is a cationic radical that accepts electrons, turns to the neutral form, and loses its characteristic bluish-green color. The antioxidant activity of Am-CS and its bio-nanocomposite was evaluated in the ABTS assay, and the results are shown in Figure 7. The results indicate that the ZnO@Am-CS bio-nanocomposite membranes demonstrated higher ABTS^•+^ radical scavenging activity than the Am-CS membrane. A significant increase in antioxidant activity was perceived with an increasing ZnONP concentration from 0.025 to 0.1% in the membrane matrix. The maximal radical scavenging of 46% was accomplished by the ZnO (0.1%)@Am-CS bio-nanocomposite sample, compared to 29% for the native Am-CS membrane sample. The increase in the antioxidant action could be described by the incidence of more electron-donating atoms (i.e., nitrogen and oxygen) in the membrane matrix, which boost its ability to scavenge ABTS^•+^ radicals [52]. In addition, ZnONPs possess antioxidant potency via various mechanisms comprising free radical scavenging and reducing activities [53,54,55].

#### 2.6.3. Evaluation of In Vitro Biodegradability

**Figure 8:**
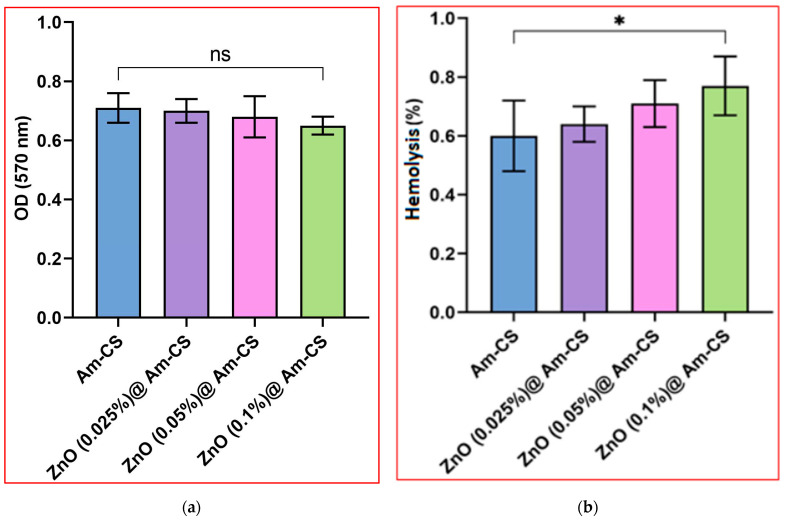
(**a**) Biodegradability and (**b**) hemocompatibility of Am-CS and its bio-nanocomposite with different ZnO concentrations. All measurements are presented in five replicates (*n* = 5), and the data are expressed as the mean standard deviation ± SD. * *p* < 0.05, and (ns) indicates a non-significant difference.

Figure 8a presents the enzymatic degradation of the pure Am-CS and its bio-nanocomposite membranes. The results reveal that all examined membrane samples were biodegradable in the presence of the lysozyme enzyme. These results could be attributed to the biodegradation nature of the original Am-CS derivative [56]. The formulated bio-nanocomposite membranes have functional hydrophilic amino and hydroxyl groups, which have an affinity for lysozyme adsorption, while the glycosidic bonds would be hydrolyzed, initiating the degradation of the membrane constituents [46,52]. However, the biodegradation rate decreased slightly with the increasing ZnONP content in the membrane matrix. These observations could be attributed to the stability of ZnONPs and the consumption of some hydrophilic functional groups of Am-CS throughout the interactions with ZnONPs.

#### 2.6.4. Evaluation of Hemocompatibility

The most common problem encountered when foreign substances are implanted into the blood is usually the fast formation of visual thrombus material on the foreign surface [57]. Hemocompatibility is vital to inspect the blood compatibility of examined bio-nanocomposite membranes for future biomedical applications. According to the American Society for Testing and Materials (ASTM) [58], biomaterials have been classified into non-hemolytic (hemolysis index < 2%), slightly hemolytic (hemolysis index 2–5%), and hemolytic materials (hemolysis index > 5%). The results imply that all bio-nanocomposite membranes were non-hemolytic, as shown in Figure 8b. Although the increase in the ZnONP concentration from 0.025 to 0.1% had a slight consequence on the hemolysis index, all values were at low and safe levels (i.e., <2%). These outcomes endorse that ZnO@Am-CS bio-nanocomposite membranes are biocompatible due to the viable biocompatibility nature of the biopolymer and the ZnONPs. These results agree with our previously reported studies [8,59].

#### 2.6.5. Evaluation of Cytotoxicity

**Figure 9:**
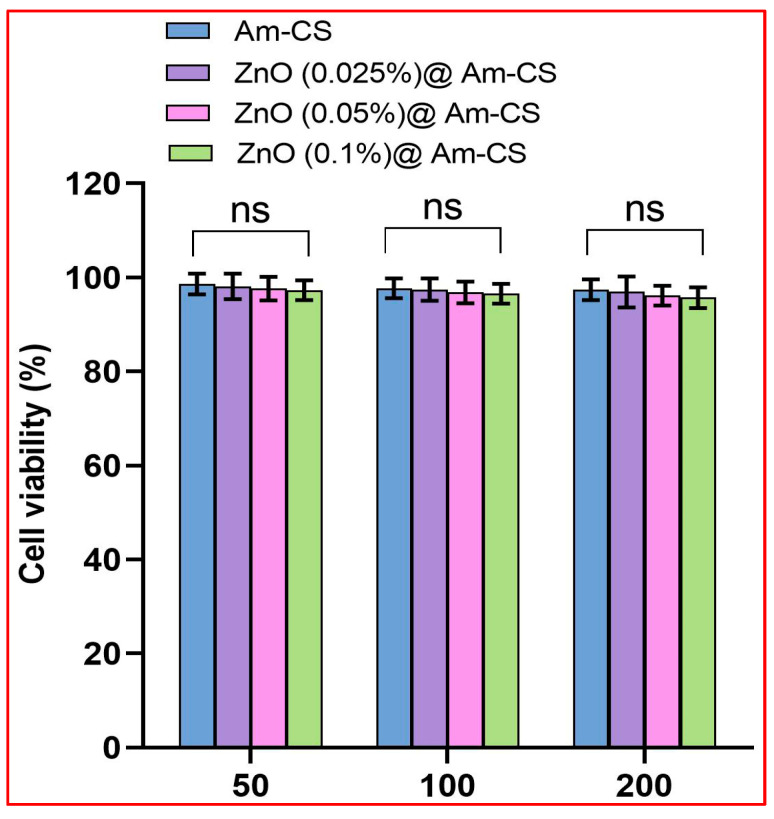
Cell viability (%) of Am-CS and its bio-nanocomposite with different ZnO concentrations. All measurements are presented in five replicates (*n* = 5), and the data are expressed as the mean standard deviation ± SD. (“ns” indicates a non-significant difference).

The MTT assay has been accepted as a potent metabolic marker to evaluate cell proliferation. As revealed in Figure 9, the cytotoxicity assay was investigated using different amounts of membrane samples (50, 100, and 200 mg). The results clarify that increasing the sample dose from 50 to 200 mg had no observable effect on the cellular toxicity. In addition, a very insignificant difference in the cytotoxicity of the ZnO@Am-CS bio-nanocomposite membranes was detected with increasing the ZnONPs from 0.025 to 0.1% in the membrane matrix. Approximately 95.7–96.9% of viable cells was perceived by the ZnO (0.1%) @Am-CS sample at the highest concentration of tested samples (200 mg). According to the international organization for standardization ISO 10993-5 [60], the materials are considered non-toxic when their toxicity is less than 25%. Accordingly, the fabricated bio-nanocomposite membranes were found to be non-toxic and acceptable for biomedical applications, specifically wound healing.

## 3. Materials and Methods

### 3.1. Materials

Chitin (acetylation degree > 95%), p-benzoquinone (pBQ; purity ≥ 94%), 3,5-Dinitrosalicylic acid (DNS; assay 98%), potassium persulfate (assay 98%), lysozyme enzyme (assay ≥ 95%), 3-(4,5-dimethythiazol-2-yl)-2,5-diphenyltetrazolium bromide (assay ≥ 97%), and dimethyl sulfoxide anhydrous (DMSO; assay ≥ 99.9%) were acquired from the Sigma Aldrich Company (Darmstadt, Germany). Zinc oxide NPs (99.8%, 50 ± 10 nm) were procured from Aladdin Chemical Co., Ltd. (Shanghai, China). Ethylene diamine (EDA; assay ≥ 94%) was imported from Oxford Lab Fine Chem. LLP. (Mumbai, India). Acetic acid (purity 99.8%), sodium hydroxide (purity 98%), and ethanol (98%) were purchased from Loba Chemie (Maharashtra, India).

### 3.2. Microorganisms

For the antibacterial activity assay, three Gram-negative bacteria were selected, namely *Escherichia coli* (*E. coli*; ATCC 8739), *Klebsiella pneumoniae* (*K. pneumoniae*; ATCC 13883), and *Pseudomonas aeruginosa* (*P. aeruginosa*; ATCC 90274), while *Staphylococcus haemolyticus* (*S. haemolyticus*; ATCC 29970) was used as a Gram-positive bacteria. The selected bacteria were refreshed through inoculation in LB broth culture medium (pH7) comprising 1% peptone, 0.5% yeast extract, and 1% NaCl. All media were incubated for 24 h at 37 °C in a shaking incubator at a constant shaking speed of 150 rpm.

### 3.3. Preparation of Amine-Functionalized Chitosan (Am-CS)

Amine-functionalized chitosan derivative was prepared according to a previously reported study [61]. In brief, an accurate amount of chitin (4 g) was dispersed in a solution of pBQ (6.9 mM, pH 9), and the reaction was conducted at 30 °C for 6 h under gentle stirring to activate the hydroxyl groups of chitin. The activated chitin was separated from the activation medium and washed numerous times using distilled water to eradicate the excess pBQ molecules. Next, the amination process was performed by dispersing the activated chitin into a solution of EDA (6.9 mM), while the reaction mixture was left for 6 h at 60 °C under continuous stirring. The resulting product was filtered and washed using distilled water to eliminate the excess unreacted EDA molecules. Finally, the formed amine-functionalized chitin was deacetylated by soaking in a solution of 50% NaOH for 6 h at 120 °C under constant stirring. The produced AmCS was separated, washed using distilled water until neutrality, and dried overnight at 50 °C.

### 3.4. Formulation of ZnO@Am-CS Bio-Nanocomposite Membranes

**Figure 10:**
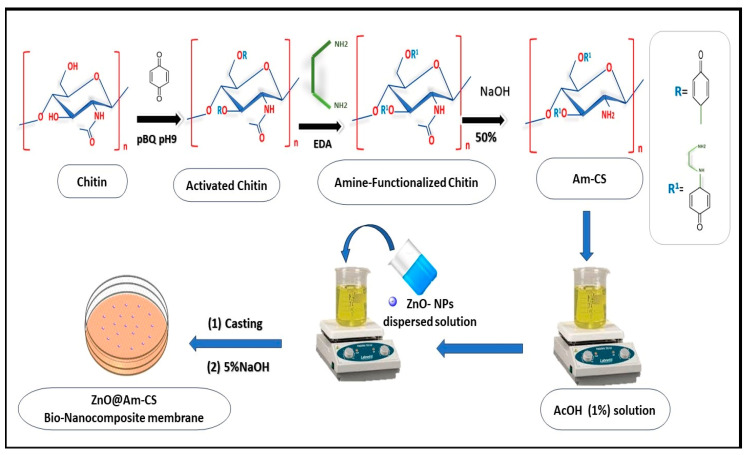
A scheme for the preparation steps of ZnO@Am-CS bio-nanocomposite membrane.

The prepared Am-CS derivative was dissolved at room temperature in acetic acid (AcOH; 1%) with a final concentration of 2% under gentle stirring until complete solubilization. Next, a proportion of ZnONPs was dispersed in distilled water and added to the Am-CS solution with a final concentration of 0.025, 0.05, and 0.1% (*w*/*v*). An accurate 0.5 mL of glycerol (as a plasticizer) was added, and the mixture was kept under continuous stirring at room temperature for 2 h. The formed bio-nanocomposite was poured into a clean Petri dish (7 cm in diameter) and stored at room temperature for 48 h. An aqueous NaOH (5% *w*/*v*) solution was poured onto the viscous membranes for neutralization and left for approximately 30 s. The wet membranes were gently separated and washed thoroughly using distilled water before being fixed onto glass plates supported with clamps. Finally, the formulated ZnO@Am-CS bio-nanocomposite membranes were dried at room temperature until they reached constant weights. The pristine Am-CS membrane was fabricated using the same procedure without adding ZnONPs. A schematic representation of the formulation process is depicted in Figure 10.

### 3.5. Instrumental Characterization

The chemical structure of the developed bio-nanocomposite membranes was inspected in the range of 4000–500 cm^−1^ using Fourier transform infrared spectroscopy (FT-IR, Model 8400 S, Shimadzu, Kyoto, Japan). The FTIR spectra were recorded using 5 mg of dried sample and KBr discs. The morphological changes were investigated using a scanning electron microscope (SEM, Joel IT200; Freising, Germany) and a transmission electron microscope (TEM, Joel JEM-100CX, Tokyo, Japan). Prior to SEM analysis, the examined membrane sample was spread on a double-sided conducting adhesive tape pasted on a metallic stub, and followed by coating with a thin gold film. In addition, energy dispersive X-ray (EDX; Oxford Instruments, Abingdon, UK) was employed to identify the elemental composition for the developed membranes. To scrutinize the mechanical properties and surface roughness of the synthesized membranes, a universal testing machine (AG-1S, Shimadzu, Kyoto, Japan) and surface roughness tester (SJ-201P, Mitutoyo, Kawasaki City, Japan) were used, respectively.

### 3.6. Water Uptake Studies

Investigation of the water uptake profile was achieved by soaking an accurate 0.1 g of the examined membrane sample in distilled water (pH 7) at room temperature until it reached equilibrium (6 h). Thereafter, the swollen sample was carefully separated and plotted between two filter papers to eradicate the excess of adhered water on the membrane surface, followed by weighing on a closed electronic balance. The water uptake percentage (WU%) was calculated according to the following equation [62].

(1) $$WU\;(\%)=\frac{W_{s}-W_{i}}{W_{i}}\times 100$$

where W*s* and W*_i_* signify the swollen and initial weights of the tested samples, respectively.

### 3.7. Antibacterial Activity Test

#### 3.7.1. Agar Well Diffusion Assay

The agar well diffusion assay was performed using the reported method [63]. In brief, 100 µL of designated bacterial suspensions were spread onto LB agar plates. The plates were left to dry for 5 min, while wells with a diameter of 5 mm were formed on the agar plate surface using a sterile cork borer. Later, the examined membrane samples were submerged in the wells, and the agar plates were incubated for 24 h at 37 °C. To estimate the antibacterial activity of the membrane samples, the diameters of the inhibition zones were measured.

#### 3.7.2. Broth Dilution Method

An antibacterial assay was carried out according to the author’s previous studies using the broth dilution method [8,49]. The previously refreshed bacterial suspensions were diluted by 100 times in 1% LB medium. Using test tubes, 0.1 mL of the diluted suspension was cultivated in 10 mL of the peptone medium, followed by adding the examined membrane sample (10 mg/mL). The bacterial culture without a membrane sample was used as a control. Thereafter, the tubes were incubated under constant shaking (150 rpm) for 24 h at 37 °C. The inhibition of bacterial growth was analyzed as a function of the optical density (OD) using visible spectroscopy (Optima SP-300, Tokyo, Japan) at 620 nm according to the following equation:

(2) $$Bacterial\;inhibition\left(\%\right)=\frac{{OD}_{1}-{OD}_{2}}{{OD}_{1}}\times 100$$

where OD_1_ and OD_2_ represent the optical densities of bacterial culture without and with the presence of the tested membrane samples, respectively.

### 3.8. Antioxidant Activity Assay

Following the reported ABTS decolorization assay [48,64], the antioxidant activity of the developed bio-nanocomposite membranes was estimated. Briefly, the radical cations of ABTS^•+^ were made by reacting 17.2 mg of ABTS with 3.3 mg/5 mL of an aqueous solution of K_2_S_2_O_8_. Next, the produced bluish-green radical cation solution was stored overnight in the dark below 0 °C. Then, 1 mL of the radical solution was diluted with 0.5% acetic acid solution to obtain a total volume of 60 mL. A definite quantity (100 mg) of the tested membrane sample was soaked in a glass test tube comprising the diluted ABTS^•+^ solution (2 mL) and subsequently incubated for 0.5 h in the dark at 25 °C. Finally, the percentage of radical scavenging was assessed at a wavelength of 734 nm using a visible spectrophotometer according to the following equation:

(3) $$Radical\;scavenging\;(\%)=\frac{A_{a}-A_{b}}{A_{a}}\times 100$$

where A_a_ and A_b_ are the absorbances of the ABTS solution in the absence and presence of the examined membrane sample, respectively.

### 3.9. In Vitro Hemocompatibility Study

The blood compatibility of the synthesized ZnO@Am-CS bio-nanocomposite membranes was studied via estimation of the hemolysis of red blood cells in the incidence of the tested membrane sample according to the American Society for Testing and Materials (ASTM) (ASTM F 756-00, 2000) [58]. Using sterile test tubes, a defined amount of membrane sample was soaked in 7 mL of PBS (pH 7) and incubated for 72 h at 37 °C. After that, the PBS was taken out, and 3 mL of citrate dextrose (ACD) solution (with 1 mL of anticoagulant and 9 mL of fresh blood) was injected into each tube. The tubes were then incubated at 37 °C for 3 h. The tubes were inverted twice each for 0.5 h to preserve contact between the examined sample and the blood. Later, each tube was centrifuged for 15 min at 2000 rpm. Using visible spectroscopy, the released hemoglobin during the hemolysis process was assessed at 540 nm as a function of the optical density (OD). Additionally, positive and negative controls were performed by adding ACD blood to 7 mL of deionized water and PBS, respectively. The following equation can express the degree of hemolysis:

(4) $$Hemolysis\left(\%\right)=\frac{{OD}_{s}-{OD}_{n}}{{OD}_{p}-{OD}_{n}}\times 100$$

where OD_s_ denotes the optical density of the supernatant in the presence of the tested sample, and OD_p_ and OD_n_ are the optical densities of the positive and negative controls, respectively.

Notably, informed consent was obtained from a volunteer (28 years old) before using his blood. In addition, all approaches were executed in accordance with the relevant guidelines and regulations.

### 3.10. In Vitro Enzymatic Biodegradability Study

The enzymatic biodegradability of the developed ZnO@Am-CS bio-nanocomposite membranes was evaluated using a dinitrosalicylic acid (DNS) reagent [65]. An exact quantity of tested membrane samples (100 mg) was immersed in test tubes containing phosphate buffer (pH 7) and lysozyme enzyme (0.5 mL) solutions. The tubes were placed for 24 h at 37 °C in a shaking water bath at a constant shaking rate of 150 rpm. Then, the activity of the lysozyme was stopped via the addition of 1.5 mL of DNS reagent, while the test tube was boiled for 15 min, and finally, left to cool at room temperature. The color produced by the reaction of the DNS reagent with the liberated reduced sugar from the membrane sample was assessed using visible spectroscopy by quantifying the optical density at 570 nm.

### 3.11. In Vitro Cytotoxicity Assay

Using fibroblast cell lines (American Type Culture Collection (ATCC, Manassas, VA, USA)), the MTT [3-(4, 5-dimethythiazol-2-yl)-2,5-diphenyltetrazoliumbromide] assay was used to measure the toxicity of the ZnO@Am-CS bio-nanocomposite membranes [66,67]. Using tissue culture plates (96 well), a complete monolayer sheet was settled after incubation at 37 °C for 24 h with 1 × 10^5^ cells/mL (100 uL). The growth medium was poured after obtaining a confluent sheet of cells. Next, the cell monolayer was washed twice with washing medium, while Roswell Park Memorial Institute (RPMI) medium containing serum (2%) was used as the maintenance medium. The cells (a normal fibroblast cell line) were treated with specific quantities of sterilized membrane samples (50, 100, and 200 mg). Moreover, three wells served as controls, while the outstanding wells simply received a maintenance medium as they were examined with 0.1 mL of each dilution, followed by incubation at 37 °C. Likewise, the cells were analyzed for any physical signs of toxicity. Specifically, 20 uL of a previously prepared MTT solution (5 mg/mL in PBS) was added to each well, followed by shaking for 5 min. To allow the MTT to be metabolized, the cells were then incubated at 37 °C in a humidified CO_2_ (5%) incubator for 1–5 h. Lastly, the medium was discarded, while DMSO (200 µL) was used to re-solubilize the formazan crystals (a metabolic agent of MTT), followed by shaking (150 rpm) for 5 min to appropriately combine the formazan and solvent. Using a spectrophotometer, the optical density was assessed at 620 nm, which was closely correlated with the cell count, as represented by the following equation:

(5) $$Cell\;viability\left(\%\right)=\frac{{OD}_{treated}}{{OD}_{Untreated}}\times 100$$

### 3.12. Statistical Analysis

All examinations were conducted in five replicates, and the data were statistically analyzed utilizing one-way analysis of variance (ANOVA) with Tukey’s analysis using GraphPad Software Inc., Version 8, San Diego, CA, USA. All results are shown as the mean ± SD and considered significant at *p* ≤ 0.05.

## 4. Conclusions

In summary, a new bio-nanocomposite membrane based on ZnONPs and amine-functionalized chitosan was formulated, with multiple features comprising antibacterial, antioxidant, biodegradable and biocompatible properties. The impacts of variation in the ZnONP content on the water uptake profile, roughness, and mechanical properties were investigated. The antibacterial activity of the ZnO@Am-CS bio-nanocomposite membrane was boosted by increasing the ZnONPs in the membrane matrix towards *E. coli* (89.4%, 38 ± 0.17 mm), *P. aeruginosa* (83.8%, 34 ± 0.42 mm) and *K. pneumoniae* (75.3%, 31 ± 0.33 mm) as Gram-negative bacteria and *S. haemolyticus* (61.4%, 25 ± 0.21 mm) as Gram-positive bacteria. In addition, the ABTS assay proved the competence of the developed bio-nanocomposite membrane in scavenging radicals, with maximal values reaching 37.2–46.6%. The enzymatic degradation study indicated that all developed membranes were biodegradable, while they established better hemocompatibility. The in vitro cytotoxicity assessment revealed the biosafety of the fabricated bio-nanocomposite membrane with cell viability in the range of 95.7 ± 2.2–96.9 ± 3.3%, inferring its future practical application as a potent antibacterial and antioxidant material for hastening the wound healing process.

## Figures and Tables

**Table 1:** The mechanical parameters of the Am-CS membrane and the ZnO@Am-CS bio-nanocomposite membrane. All measurements are presented in five replicates (*n* = 5), and the data are expressed as the mean standard deviation ± SD.

Membrane	Max. Force (N)	Max. Stress σ_max_ (N/m^2^)	Max. Strain ʎ_max_ (%)
Am-CS	120.2 ± 1.3	42.1 ± 2.1	6.3 ± 1.1
ZnO (0.025%) @Am-CS	128.4 ± 1.2	53.4 ± 1.7	10.4 ± 1.3
ZnO (0.05%) @Am-CS	136 ± 1.4	59.2 ± 1.5	15.4 ± 2.1
ZnO (0.1%) @Am-CS	147 ± 1.2	64.4 ± 1.3	17.5 ± 1.5

## Data Availability

The data presented in this study are available upon request from the corresponding author.

## Sample Availability

Samples are available from the authors.
